# Engineered protein nanofibrils for chain-integrated water treatment: From source monitoring to end-point purification

**DOI:** 10.1016/j.eehl.2026.100273

**Published:** 2026-08-24

**Authors:** Yiwei Liu, Zhengzheng Zhao, Yang Wu, Min Long, Xiong Zheng, Yinguang Chen

**Affiliations:** a State Key Laboratory of Water Pollution Control and Green Resource Recycling, College of Environmental Science and Engineering, Tongji University, Shanghai, 200092, China; b Key Laboratory of Yangtze River Water Environment, College of Environmental Science and Engineering, Tongji University, Shanghai, 200092, China; c Shanghai Institute of Pollution Control and Ecological Security, Shanghai, 200092, China; d Shanghai Key Laboratory of Urban Renewal and Spatial Optimization Technology, Shanghai, 200092, China

**Keywords:** Protein nanofibrils, Surface modifications, Functional nanoparticles, Contaminant adsorption, Biosensors, Membrane coatings

## Abstract

Escalating complexity in water pollution has catalyzed a shift toward chain-integrated management, encompassing source monitoring, targeted removal, and end-point purification. Protein nanofibrils (PNFs), self-assembled from abundant proteins, are ideal candidates for these challenges due to their rich functional groups, mild synthesis, and tunability. This review examines PNFs’ potential in chain-integrated water treatment technologies. First, the evolution of PNFs reveals their advantages over conventional nanomaterials such as cellulose nanofibrils and carbon nanotubes, including simpler preparation methods, superior physicochemical properties, and versatile functionalization capabilities. Second, PNFs serve multifunctional roles across the water treatment chain: as sensitive biosensor components for contaminant detection, selective adsorbents for pollutant removal, stable catalyst supports for degradation reactions, and effective membrane modifiers for filtration enhancement. The exceptional performance of PNFs results from abundant functional groups and controllable surface charge, which provide binding sites for contaminants and attachment points for functional materials, as well as their high aspect ratio that prevents nanoparticle aggregation while maintaining catalytic efficiency. Despite their commercial emergence as adsorbents, PNF-based systems face key bottlenecks, including limited biosensing selectivity, elusive adsorption mechanisms, and inadequate stability for catalysis. Future research directions should focus on developing interference-resistant binding strategies, optimizing macrostructure preparation, creating stable catalytic systems, and advancing structural analysis through molecular engineering approaches. This comprehensive review provides insights into these novel biomaterials and their potential in next-generation water treatment technologies.

## Highlights

• PNFs serve as sustainable alternatives to nanomaterials in water treatment.
• Strong PNF-pollutant affinity enables accurate contaminant detection.
• Inherent functional groups of PNFs ensure effective pollutant adsorption.
• PNFs control nanoparticle size to enhance composite performance.
• Future work should focus on maintaining fibril stability and functionality.

## Graphical abstract

**Figure:**
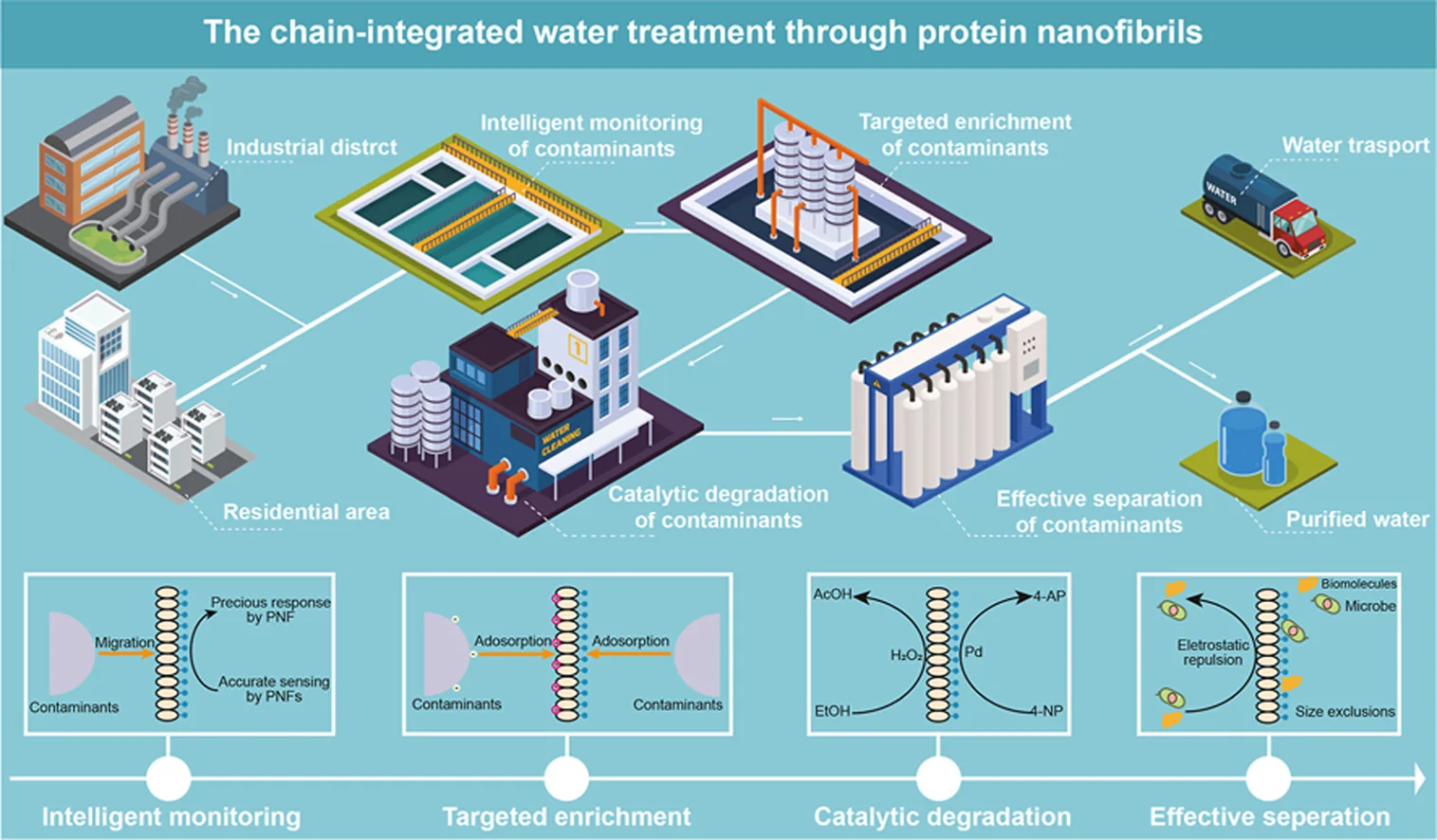

## 1 Introduction

The global water environment faces unprecedented challenges due to rapid urbanization, emerging contaminants, and climate change [1]. Urban population growth intensifies the imbalance between water supply and demand, placing substantial pressure on urban water systems. Projections indicate that the urban population experiencing water scarcity will increase from 933 million in 2016 to 1.693–2.373 billion by 2050 [2]. Water pollution has evolved from single-contaminant issues to complex scenarios involving multiple pollutants, with researchers identifying potential risks from 473 distinct compounds in aquatic ecosystems [3]. Climate change further compounds these challenges by amplifying existing spatial and temporal disparities in water resource distribution. According to the WHO, nearly half of the global population will experience water stress by 2025. These compounding factors expose the limitations of conventional water treatment technologies, emphasizing the critical need to develop innovative water treatment solutions.

**Fig. 1:**
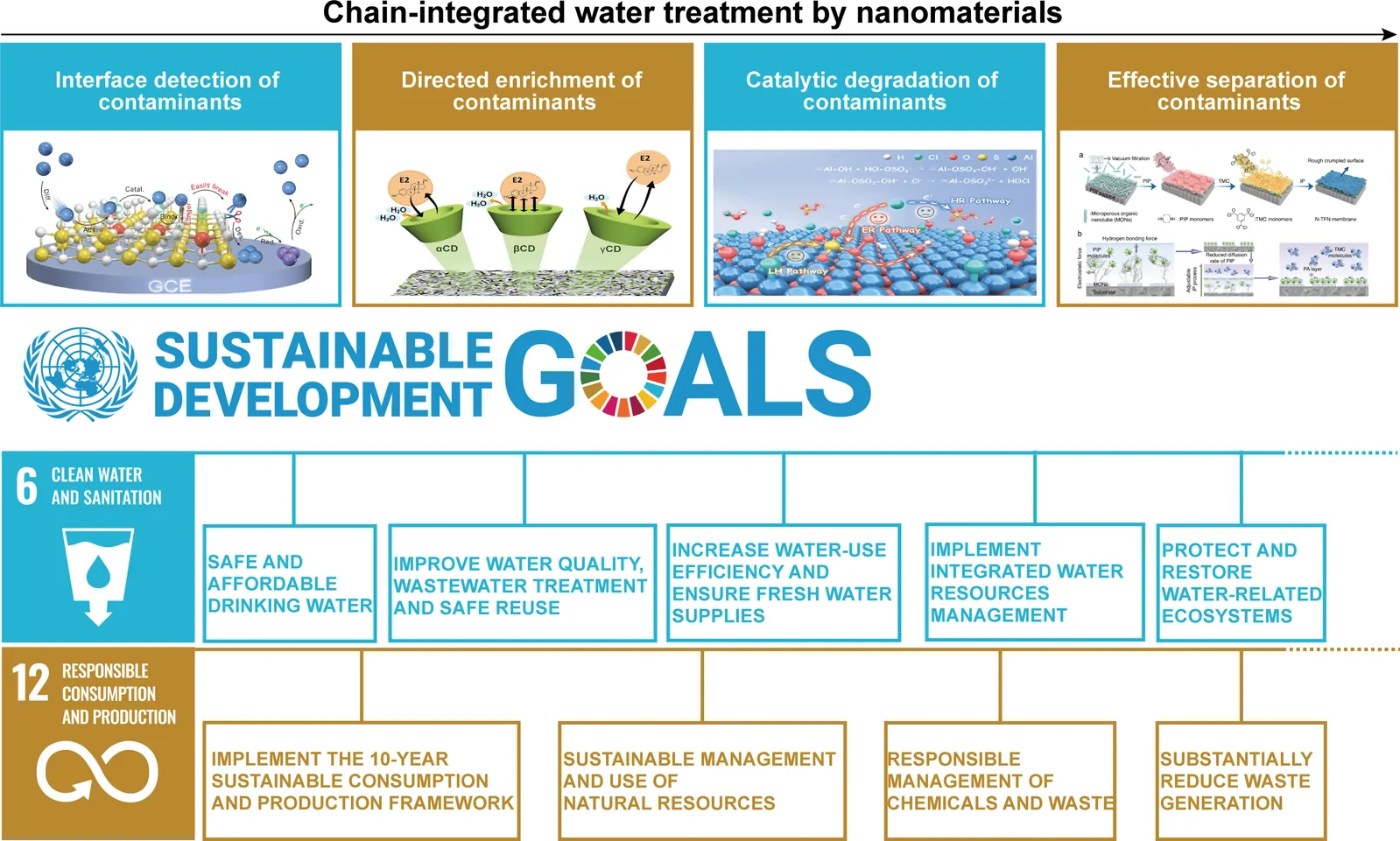
The overview of the chain-integrated water treatment processes by nanomaterials, including contaminant detection, targeted adsorption, catalytic degradation, and membrane separation, adapted from Refs. [[4], [5], [6], [7]], and the role of clean water and sanitation (SDG 6) and responsible consumption and production (SDG 12) in achieving other SDGs, adapted from Ref. [12]. Copyright American Chemical Society ([4,5]), Springer Nature ([6]) and Elsevier ([7]).

Advances in nanotechnology offer promising solutions to address the complex challenges in water environment management. As illustrated in Fig. 1, these developments have transformed water treatment from single-process approaches to integrated treatment systems that combine interface detection, targeted enrichment, catalytic degradation, and contaminant separation [[4], [5], [6], [7]]. However, nanomaterial applications raise significant environmental concerns [8]. The production of nanomaterials often relies on non-renewable resources and requires complex, energy-intensive processes involving high temperatures and hazardous chemicals [9]. Additionally, the increasing deployment of nanomaterials risks releasing harmful chemical byproducts into the environment, raising questions about their long-term sustainability [10,11]. These concerns align with the United Nations’ Sustainable Development Goals (SDGs) 6 (clean water and sanitation) and 12 (responsible consumption and production), which emphasize the integration of water quality management with sustainable societal and economic development by 2030 (Fig. 1) [12]. Consequently, research attention has shifted toward bio-based materials, which offer advantages in efficiency, renewability, and biocompatibility.

Protein nanofibrils (PNFs) represent promising candidates for integrated water treatment systems, owing to their unique structural properties and versatile applications. Their exceptional aspect ratio and tunable macrostructures enable efficient capture of diverse contaminants [13]. The inherent chemical groups and residues of PNFs confer remarkable adsorption capabilities through zwitterionic and amphiphilic properties [14]. PNFs-based adsorbents demonstrate high removal efficiencies (e.g., exceeding 90% for heavy metals and 99% for radioactive pollutants) through surface group interactions [15]. Beyond their primary binding functionality, these surface groups can undergo chemical modifications to create molecular-level recognition sites and catalytic centers for pollutant degradation. This versatility has been demonstrated in environmental applications through composite adsorbents, catalytic scaffolds, and sensing platforms [16,17]. The cross-β-sheet arrangements in PNF microstructures provide exceptional nanomechanical stability and resistance to enzymatic degradation [18]. In practical applications, PNF-based adsorption membranes maintain complete contaminant removal during seven months of continuous operation [19]. While existing reviews have primarily concentrated on the preparation strategies of PNFs and their potential for contaminant adsorption [14,20,21], a systematic analysis of their roles in integrated water treatment processes is currently lacking, particularly in interface detection, targeted enrichment, catalytic degradation, and contaminant separation.

This review introduces a novel chain-integrated water treatment model based on PNFs and examines their applications in water purification systems. The work begins with an exploration of the rationale for PNFs in environmental engineering, addressing their development trajectory, synthesis methods, and comparative advantages over conventional cellulose-based materials. The review then summarizes the relationships between PNF properties and their diverse applications in water treatment, including their roles as adsorbents, catalytic building blocks, and pollutant sensors. Moreover, the discussion concludes with an examination of current limitations, emerging challenges, and strategic directions for PNF development. This review highlights the potential of biomaterials for water treatment applications.

## 2 Development and manufacturing strategies of PNFs for water treatment

### 2.1 Evolution from pathological structures to functional materials

**Fig. 2:**
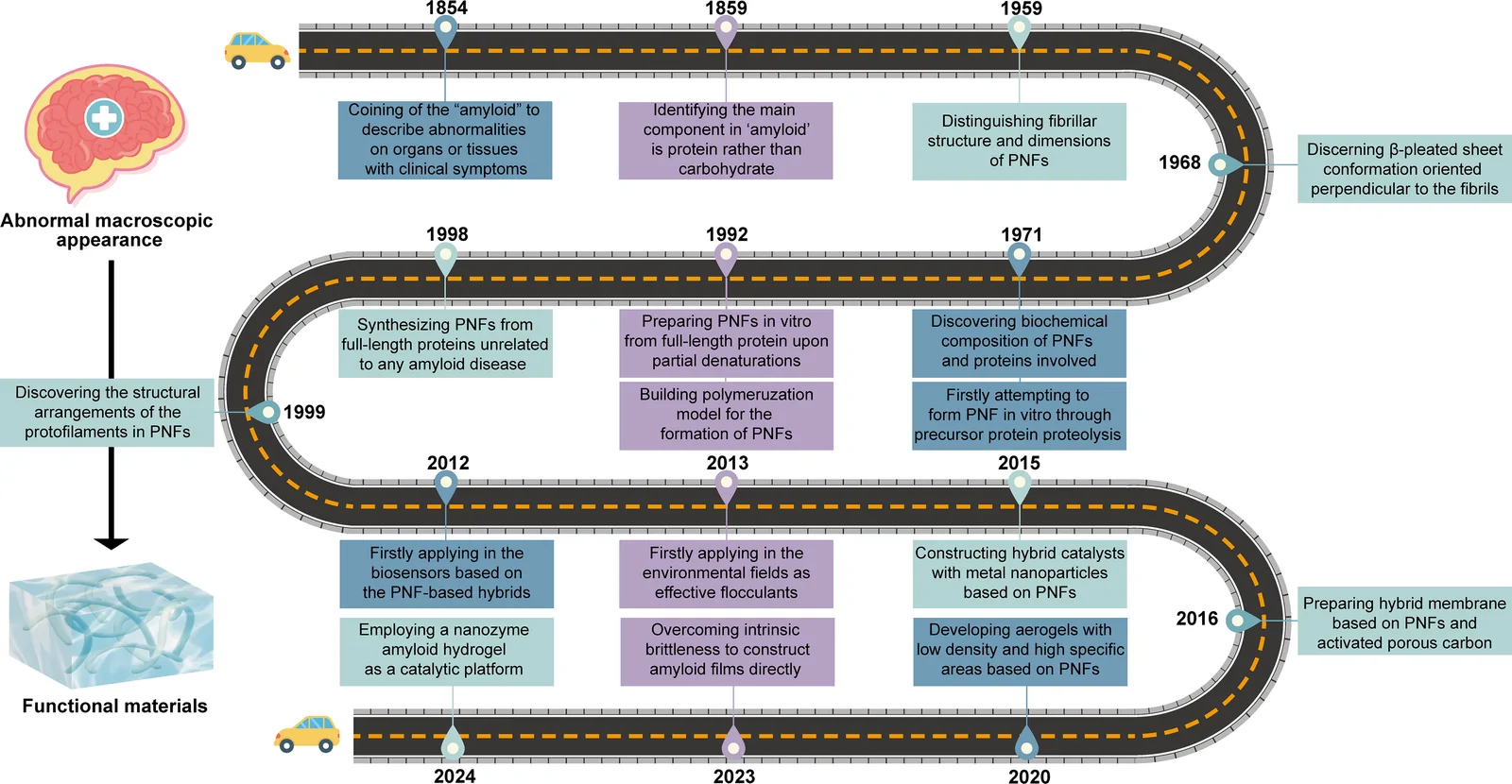
Timeline of key advances in PNFs in environmental applications, including their initial discoveries, structural identification, component analysis, in vitro preparation, applications as flocculants, adsorbents, and catalysts.

The understanding of PNFs has shifted from their initial recognition as pathogenic amyloids to their current status as functional materials [22] (Fig. 2). The concept of amyloid was initially introduced in 1854 by German physician Rudolph Virchow, who employed primitive iodine staining to examine cerebral corpora amylacea exhibiting abnormal macroscopic characteristics [22]. Throughout the 19th and early 20th centuries, PNFs research evolved from macroscopic observations to clinical classifications, revealing that these structures consisted primarily of protein and displayed consistent fibrillar characteristics with perpendicular β-pleated sheet conformations, regardless of their origin [23,24]. Subsequent investigations determined their biochemical composition and protein identity through amino acid composition and protein sequence analysis [25]. The distinctive structure of these fibrils garnered substantial scientific interest, leading to attempts at in vitro preparation of amyloid fibrils through precursor protein proteolysis [26]. In 1992, a notable achievement was the successful conversion of a full-length amyloidogenic protein with partially denatured quaternary and tertiary structures into PNFs, alongside the establishment of a nucleation-dependent polymerization model for PNF formation [27,28]. A paradigm shift occurred in 1998 when research suggested that amyloid deposition might represent a common property of proteins rather than being exclusively associated with pathological conditions [29]. The following year, cryo-electron microscopy enabled the proposal of a polypeptide packing model, establishing a fundamental framework for understanding general amyloid fibril structure [30]. Contemporary research has demonstrated that these specific structural characteristics confer remarkable stability [18], establishing a foundation for diverse applications.

Since the initial report of PNF-based hybrid biosensors in 2012, these materials have served as a cornerstone for further technological progress [18]. Meanwhile, heavy metals can trigger PNF formation in neurodegenerative diseases through peptide binding, suggesting potential applications in contaminant removal [31]. This insight led to environmental applications of PNFs, beginning with their development as eco-friendly flocculants for toxic elements and organic contaminants in 2013 [32]. As fundamental supramolecular units [33], PNFs have enabled various macroscopic materials, including hybrid membranes with activated porous carbon that effectively remove heavy metals and radioactive waste from water [15]. Between 2016 and 2020, both pure PNF aerogels and hybrid composites demonstrated significant adsorption capacity for heavy metals and organic pollutants [34]. The metal-binding affinity of PNFs enables novel composite synthesis, particularly through biomineralization of metal ions into nanoparticles [35]. In 2015, PNFs served as templates for gold and palladium nanoparticle synthesis, leading to hybrid composites for environmental catalysis [36]. These PNF-nanoparticle combinations have expanded water treatment capabilities, functioning as hybrid adsorbents, catalysts, and biosensors [16,17]. Over the past three years, researchers have overcome the intrinsic brittleness of these materials, enabling the fabrication of macroscopic and tunable amyloid films [37]. Furthermore, single-site iron-anchored amyloid hydrogels have been validated as catalytic platforms using X-ray absorption fine structure and X-ray absorption near-edge spectroscopy [38]. Recently, the feasibility of fabricating protein materials via large-scale self-assembly has been demonstrated, significantly broadening their potential applications [39].

### 2.2 Manufacturing strategies and composite development

#### 2.2.1 Conventional and accelerated strategies for PNF formation

Recent research has focused on strategies to produce functional PNFs. As shown in Table S1, multiple conditions can initiate PNF formation, including chemical denaturation with chaotropic agents, disulfide bond modification, low pH, and elevated temperatures. The conventional approach involves protein incubation at specific temperatures under acidic conditions, triggering a sequence of protein unfolding, misfolding, hydrophobic aggregation, and fibril transition [18]. During this process, acidic conditions facilitate protein hydrolysis into smaller peptides that self-assemble in an ordered manner, while elevated temperatures accelerate assembly kinetics [40]. However, the required incubation time varies significantly among protein types and presents a major challenge for scalable production. For instance, soy protein isolate forms PNFs within 8 h, whereas lysozyme requires extended incubation periods exceeding 24 h under identical conditions [41]. These variations in incubation time reflect differences in protein properties and their corresponding hydrolysis rates.

Inefficient denaturation remains a major challenge in conventional PNF production through amyloid aggregation. Recent advances have introduced various methods to accelerate PNF formation beyond traditional approaches, including microwave heating and salt addition to protein solutions [42]. Specifically, NaCl and CaCl_2_ enhance PNF growth rates by reducing electrostatic repulsion and promoting aggregations [43]. Chemical agents such as ionic liquids, deep eutectic solvents, and protein denaturing agents have also emerged as effective catalysts for PNF formation [[44], [45], [46]]. Chaotropic agents, in particular, significantly expedite lysozyme fibrillation compared to the conventional method [44]. For globular proteins with high fibrillation propensity, abundant α-helix structures, and intramolecular S−S bonds, tris(2-carboxyethyl)phosphine (TCEP) can induce amyloid aggregation within minutes by disrupting these structural elements [47]. However, these acceleration methods can alter PNF characteristics: salt solutions affect viscosity and mechanical properties, while the reduction of agent-induced aggregation produces distinct protofibrils through nucleation-dependent monomer unfolding [47].

#### 2.2.2 Supramolecular assembly and morphology preservation

**Fig. 3:**
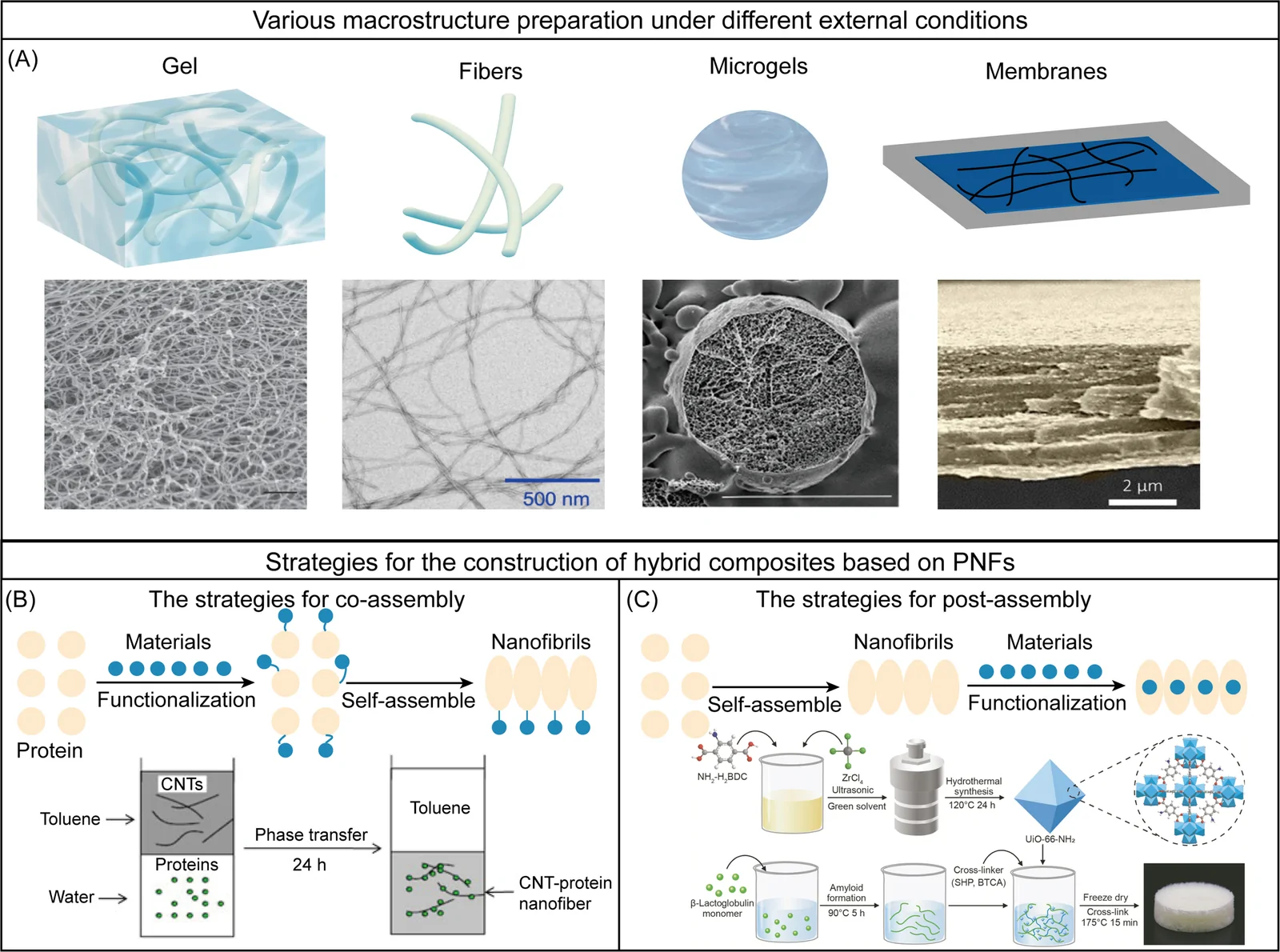
Overview of the various macrostructures based on PNFs (A) and the schematic illustration of the strategies for the construction of hybrid composites based on PNFs, including co-assembly between PNFs and carbon nanotubes (B) and post-assembly between PNFs and UIO-NH_2_-66 (C). Electron micrographs adapted from Refs. [48,[50], [51], [52]]; co-assembly and post-assembly diagrams adapted from Refs. [58] and [59], respectively. Copyright American Chemical Society ([48,52]), Wiley-VCH ([50]),American Association for the Advancement of Science ([51]), Elsevier ([58]) and Royal Society of Chemistry ([59]).

As fundamental supramolecular units, PNFs can assemble into diverse macroscopic materials, ranging from one-dimensional elongated fibers and two-dimensional integrated membranes to three-dimensional structures such as continuous bulk gel networks and discrete, micron-sized microgel particles (Fig. 3A) [13,15,33,[48], [49], [50], [51]]. The distinct physicochemical properties and environmental applications of these forms are governed by their specific processing strategies, such as gelation, microfluidics, and vacuum filtration [52,53]. The differences in their properties enable different applications in the environmental field. For instance, lysozyme-based fibrils exhibited exceptional turbidity removal across a broad pH range due to their high linear surface charge [48]. Similarly, β-lactoglobulin PNF-derived aerogels, produced through freeze-drying, show promising water treatment potential due to their low density, large open pores, and high specific surface area. However, the inability to preserve fibril morphology limits molecular understanding of contaminant-aerogel interactions [33]. Previous studies have indicated that freeze-drying often induces the collapse of PNF networks, resulting in the formation of microcrystals that dominate the final aerogel structure rather than the original polymer networks. In contrast, supercritical drying largely preserves the native fibril morphology [54]. Moreover, preserving a stable fiber morphology maintains an extreme aspect ratio, which enhances the removal of contaminants [48]. To preserve fibrous structures, researchers have explored membrane preparation through casting, vacuum filtration, and dialysis [15,55]. These stable, ordered supramolecular structures exhibit high contaminant capture efficiency and reusability with minimal energy requirements [56]. While recent research has begun investigating nanostructure effects in protein aerogels for organic pollutant removal [57], direct evidence linking fibrous structure preservation to contaminant removal efficiency remains limited.

#### 2.2.3 Functionalization strategies and composite materials

PNFs serve as promising building blocks for bioinspired materials, owing to their high aspect ratios. Whether used independently or combined with nanocomposites, they generate functional biomaterials with diverse water treatment applications, including adsorbents, catalysts, and biosensors [34,36,60]. PNF functionalization typically follows two main approaches: co-assembly (Fig. 3B) and post-assembly (Fig. 3C), with methods carefully selected to preserve PNFs’ inherent properties [58,59,61]. As illustrated in Table 1 [60,[62], [63], [64], [65], [66], [67], [68], [69], [70], [71], [72], [73]], different materials interact with PNFs through distinct binding mechanisms, yielding varied functional properties. For instance, the incorporation of cellulose nanofibrils, montmorillonite nano-clay, or chitosan has been reported to enhance the structural strength of PNFs through electrostatic interactions with their surface functional groups. Similarly, PNF-metal nanoparticle composites form through several mechanisms, including amino acid reduction, supramolecular coordination bonds, and alkali precipitation [16,65]. The high aspect ratio of PNFs helps stabilize metal nanoparticles, control their size distribution, and prevent aggregation, thereby maintaining their catalytic activity [64]. Carbon materials serve as effective substrates for PNF composite membranes, improving their mechanical strength and hydrophilicity for enhanced performance in complex environments [15,74]. Optimizing these materials for green production and expanded functionality requires careful consideration of regulatory, modification, and incorporation strategies during synthesis. These characteristics position PNFs as promising candidates for next-generation water treatment materials.

**Table 1:** Integration of PNFs with diverse functional materials.

Types of functional materials	Types of PNFs	Combined methods	Combined mechanisms	Advantages	Ref.
Nanocellulose	Lysozyme	Magnetic agitation and vacuum filtration	-	Enhancement of thermal stability and mechanical properties	[66]
Nanocellulose, polydopamine	β-lactoglobulin	Homogenization and freeze-drying	Self-polymerization of polydopamine on nanocellulose and then reaction with groups of PNFs	-	[73]
Cellulose nanofibers	Lysozyme	*In situ* synthesis of PNFs on cellulose nanofibrils	Electrostatic interactions between PNFs and cellulose nanofibers	Excellent stability and mechanical properties	[68]
Chitosan, carbon	β-lactoglobulin	PNFs/Carbon membrane through vacuum filtration immersed in chitosan	-	Decreased pore size and increased flow rate	[72]
g-C_3_N_4_	Lysozyme	Mixing g-C_3_N_4_ with poly(ethyleneimine) and incubation	The adsorption behaviors of PNFs for g-C_3_N_4_	Pore volume: increased by 12.5-fold; BET surface area: increased from 4.5 to 39.6 m^2^/g	[69]
Activated carbon	Whey protein	Mixture solutions of PNFs and activated carbon prepared through vacuum filtration	Chemical interactions (amide group and C−O bonds) and physical interactions (electrostatic, van der Waals, steric, and solvation forces by amino acids and polypeptide chain) between PNFs and activated carbon	Taking advantage of PNFs’ properties, avoiding their aggregation, and enhancing their mechanical properties and surface area	[71]
Graphene oxides	*Bombyx mori* silk fibroin	Dispersed graphene oxide solutions through ultrasonication and then incubation with PNFs	Graphene oxides-induced silk fibroin self-assembly into nanotopographies; PNFs-assisted reduction of graphene oxides	Improved mechanical properties that are beneficial for cell adhesion	[62]
Graphene	β-lactoglobulin	Mixture of PNFs and graphene by vacuum filtration	Electrostatic aggregation of PNFs and graphene and preferential binding of broken PNFs on graphene surfaces	Higher conductivity, permeability, and more stable mechanical properties	[60]
Multiwalled carbon nanotubes (MWNTs)	β-lactoglobulin	Sulfonated MWNTs then undergo complex formation with PNFs	Electrostatic interactions between the sulfonate of functionalized MWNTs and the amino groups of PNFs	Higher thermal stability, storage modulus	[70]
Fe_3_O_4_	β-lactoglobulin	A mixture of β-lactoglobulin monomers and Fe_3_O_4_ nanoparticles followed by incubation in an acidic and thermal environment	Electrostatic interactions between negative nanoparticles and positive groups of PNFs	Introduction of magnetic properties and extension of their applications	[63]
Gold nanocluster	Bovine serum albumin (BSA)	Vigorous stirring of the mixture of HAuCl_4_ and BSA solutions, adjustment of pH through NaOH, then mixture with graphene	BSA as a reducing agent and stabilizer; reduction of Au^3+^ into Au nanoparticles through SH and phenolic groups of PNFs; the combination relies on electrostatic and π−π interactions stabilizing the combination	Longer fiber structures, higher stability, and detection and separation functionality	[67]
ZrO_2_	β-lactoglobulin	Shaking of ZrOCl_2_∙7H_2_O and PNFs and then adjustment of pH to achieve deposition in situ	Electrostatic interactions between the Zr precursor and the charged amino acids of PNFs	Increased PNFs diameters and the regulation of the particle size of ZrO_2_ nanoparticles to guarantee their activities	[65]
Pd nanoparticles	β-lactoglobulin	PNFs were bound with carbon papers and then immersed in the solution of Na_2_PdCl_4_, and subsequently NaBH_4_ was added into the solutions, incubated, and dried	Electrostatic, hydrophobic, and hydrogen-bonding interactions between carbon paper and PNFs, and interactions between -NH_2_ of PNFs and Pd nanoparticles	Increased PNFs diameters, enhanced nano-Pd loadings, and larger catalytically active area	[64]

## 3 Multifunctional applications of PNFs in chain-integrated water treatment

### 3.1 Intelligent monitoring of contaminants by protein nanofibrils

Precise and reliable monitoring of aquatic contaminants plays a crucial role in assessing water quality and public safety risks [75]. Biosensors based on whey PNFs have emerged as promising detection platforms owing to their chemical versatility and adaptable functionalization capabilities [17]. The fibrillar architecture of PNFs serves as an effective scaffold for immobilizing various sensing components, including enzymes, antibodies, graphene, and nanoparticles [17]. Their inherent affinity for diverse contaminants makes them particularly suitable for water quality monitoring applications. These biosensors operate through detecting conformational changes in amyloid structures upon analyte binding, generating optical, electrochemical, or mechanical signals that enable real-time detection and quantification [76]. PNFs derived from various protein sources have demonstrated broad sensing capabilities across different target materials. Notably, these systems can function as portable detection devices, eliminating the need for specialized personnel required in conventional analytical methods [77].

The development of PNF-based biosensors leverages their extraordinary properties, including specific enzyme and functional material affinities, facile modification potential, high surface-to-volume ratio, and diverse macrostructural features. Early studies demonstrated the successful functionalization of PNFs with multiple enzymes, specifically glucose oxidase and horseradish peroxidase, to create nanostructured glucose biosensors with broad potential applications [78]. Significant advances have been made through various functionalization strategies. For instance, biotinylated PNFs, prepared using succinimide-based biotin conjugates, effectively interact with streptavidin-modified nanoparticles, quantum dots, and glucose oxidase. These systems enable glucose monitoring via cyclic voltammetry, capitalizing on the nanoscale dimensions of whey PNFs that provide high functionalization density through their extensive surface area [17]. Further developments include hybrid sensors incorporating 2D graphene oxide nanosheets and 1D lysozyme PNFs as scaffolds for Au nanoparticles and enzymes. These systems achieve precise glucose detection within the 0.3–15 mM range, benefiting from enhanced electrochemical response due to increased electrode surface area and improved electron transfer kinetics [79]. Notably, α-synuclein PNF-immobilized enzymes demonstrate up to four-fold activity enhancement compared to their free counterparts, validating their effectiveness as sensing platforms [80]. Initially inspired by biomedical applications, PNF-based sensors have expanded into environmental monitoring, driven by their biocompatibility and versatile functionality.

Research on PNF-based biosensors remains in its early stages, primarily focusing on the detection of pH, metal ions, and organic pollutants [21]. The efficacy of PNF-based sensors stems from their inherent properties, including specific ion/contaminant affinities, adaptable modification potential, and zwitterionic properties. A notable advancement in this field involves hybrid materials combining oat plant PNFs with Au nanoparticles, which exhibit enhanced electrical conductivity and demonstrate promise for high-sensitivity sensing applications [81]. These organic-inorganic nanocomposite films enable water sensing through distinctive spectral responses, where the resonance peak undergoes a blue shift and becomes more pronounced upon water exposure due to water’s lower refractive index compared to protein. The resonance peak position also varies with pH changes in the rinsing solution; for instance, a red shift occurs when the rinsing pH approaches the isoelectric point of β-lactoglobulin, attributed to enhanced optical coupling between gold platelets. The resulting composite, exhibiting both fluorescence and surface plasmon resonance features, demonstrates particular effectiveness for pH sensing in solution-based applications [82].

In contaminant detection applications, PNFs’ capture capacities play a crucial role in sensor performance. As shown in Fig. S1A, and a notable example is the development of a colorimetric nanosensor utilizing lysozyme PNFs as metal binders and acidified diphenylcarbazide as the colorimetric probe for chromium detection without pretreatment. These hybrid biosensors, featuring surface-charged PNF nanotubes, function as effective metal-analyte binders through electrostatic attractions, achieving chromium detection limits as low as 30 ppb [83]. The robust affinity of PNF-based biosensors extends to various solutions, as demonstrated by novel hybrid membranes composed of gold nanocluster-embedded BSA PNFs and graphene oxides (GO-AuNC@BSA) for mercury ion detection. These sensors operate through decreased fluorescence intensity corresponding to increased mercury concentrations, achieving an impressive detection limit of 0.0238 nM, attributed to both mercury-cysteine residue binding and mercury Au^+^ interactions [67]. Compared to traditional sensor matrices, PNF-based sensors demonstrate superior performance in response time, sensing efficiency, sensitivity, and contaminant selectivity [84]. However, sensor performance significantly depends on protein type and fiber length characteristics, as illustrated by Fig. S1B. For instance, β-lactoglobulin shows enhanced conductivity and contaminant-sensing performance compared to bovine serum protein due to higher positive charge density, while fiber length directly influences adhesive properties, making these parameters crucial for overall sensing performance [84,85]. In general, PNFs’ inherent properties enable their widespread application in sensor technology. Their intrinsic functional groups serve as binding sites for functional materials, enabling responsive behavior to environmental changes, while their specific affinities and capture capabilities ensure effective contaminant adsorption, thereby enhancing sensitivity. The overall sensing performance is primarily determined by both the protein properties and the mean fibril length characteristics.

### 3.2 Targeted enrichment of contaminants through protein nanofibrils

**Fig. 4:**
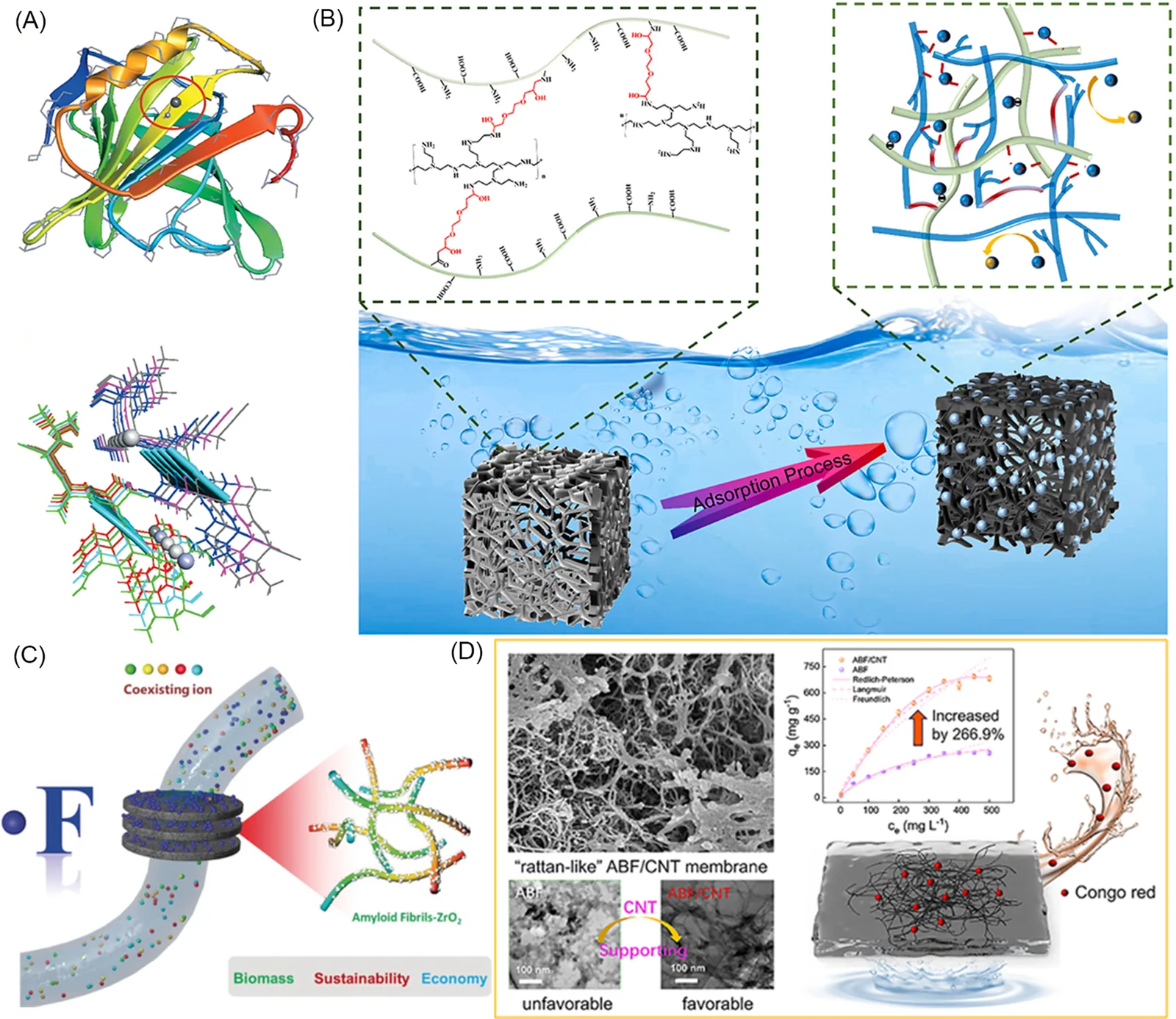
Overview of adsorption applications of PNFs. (A) Molecular structure of β-lactoglobulin with a lead ion coordinated at the Cys121 binding motif, adapted from Ref. [15]. (B) Schematic representation of the enhanced adsorption mechanism onto amino-functionalized BSA amyloid nanofibril aerogel, adapted from Ref. [89]. (C) Selective adsorption performance of the fluoride ion through β-lactoglobulin nanofibrils and ZrO_2_ hybrid membrane toward fluoride ions in the presence of competing anions, adapted from Ref. [65]. (D) Enhanced adsorption performance after integrating carbon nanotubes and BSA nanofibrils, adapted from Ref. [90]. Copyright Springer Nature ([15]), Wiley-VCH ([65]), Elsevier ([89]) and American Chemical Society ([90]).

PNFs demonstrate versatile contaminant adsorption capabilities through their various functional groups and residues, effectively capturing heavy metals, radioactive materials, and organic contaminants (Fig. 4). For instance, molecular docking studies confirmed strong binding between lead ions and Cys121 residues, as illustrated in Fig. 4A [15]. In parallel, cysteine residues provide molecular chelation sites for heavy metals, while tyrosine and tryptophan donate π-electrons to facilitate π−π stacking interactions with organic contaminants. Furthermore, the diversity of surface residues endows PNFs with zwitterionic and amphiphilic properties. Specifically, the protonated amino groups in lysine and the deprotonated carboxyl groups in glutamic acid serve as critical electrostatic binding sites [13]. Furthermore, the binding affinities are influenced by the ligand monolayer, as well as electrostatic and hydrophobic interactions through molecular dynamics simulations and density functional theory calculations [15,86]. The binding capability extends to various heavy metals, including chromium, nickel, silver, platinum, and copper [87,88]. Additionally, β-lactoglobulin PNF aerogels have shown remarkable adsorption performance for organic contaminants, achieving 98% removal of ibuprofen, 92% of bentazone, and 78% of bisphenol A, with respective adsorption capacities of 54.2, 50.6, and 69.9 mg/g. Besides, the regeneration of the aerogel over multiple consecutive cycles exhibited high reusability with no significant changes in its removal performance. This effectiveness stems from multiple synergistic mechanisms, including hydrophobic interactions, π−π stacking interactions, electrostatic interactions, and specific local interactions [33]. Moreover, the adsorption performance is governed by morphology, with straight fibrils consistently outperforming curved ones. These morphological differences were primarily determined by the specific preparation techniques employed [57].

Beyond their intrinsic adsorption properties, PNFs’ performance can be enhanced through strategic modifications during synthesis. High pH conditions during preparation expose additional amino acid residues and concentrate them on the nanofibril surface, resulting in superior lead ion capture compared to conventional methods [91]. Surface modification significantly enhances adsorption efficiency. For instance, BSA PNF aerogels functionalized with branched polyethyleneimine exhibited a 2.1-fold increase in adsorption capacity compared to their pristine counterparts, a performance boost attributed to strengthened electrostatic interactions (Fig. 4B) [89]. Similarly, the modification of nanofibrils with polyethyleneimine (PEI) resulted in a significant enhancement in lead adsorption capacity, representing an approximately 2-fold increase compared to unmodified fibrils [92,93]. However, modification outcomes largely depend on the source protein and fibril morphology. For instance, when aminosilane-modified PNF aerogels were prepared from β-lactoglobulin, lysozyme, and black bean proteins for CO_2_ capture, the black bean-derived aerogels showed notably lower adsorption efficiency due to their short, curved fibrils, which impeded silica network formation during aminosilane processing [94].

Furthermore, PNFs can be functionalized with various materials, including metal-organic frameworks (MOFs), carbon materials, metal oxides, and macromolecules, to create enhanced composite adsorbents [34,41,95,96]. For instance, hybrid aerogels combining β-lactoglobulin PNFs and ZIF-8 demonstrate exceptional environmental stability through mutual protection and reinforcement mechanisms [34]. Such functional combinations effectively address specific adsorption limitations. While PNFs’ hydrophobic amino groups effectively capture long-chain PFAS through hydrophobic interactions, they perform poorly with short-chain PFAS. This limitation is overcome by incorporating an activated carbon matrix, which improves short-chain PFAS adsorption through enhanced permeability and surface area [74]. Similarly, while unmodified β-lactoglobulin PNFs show no viral binding capacity, incorporating iron oxyhydroxide nanoparticles creates a positively charged surface that effectively captures negatively charged viruses, significantly reducing both total and infectious viral loads [97]. Selective contaminant removal presents another key challenge. ZrO_2_-modified PNF-carbon membranes achieve highly selective fluoride ion adsorption with a distribution coefficient of 6820 mg/L (Fig. 4C) [65]. Additionally, the practical viability of PNF-based hybrid membranes is underscored by their enhanced reusability and performance, which exceed those of widely used commercial alternatives like cellulose and activated carbon [15]. Currently, the applications of PNFs as adsorbents in water purification have transitioned from laboratory research to successful commercialization. A pioneering example of this technology transfer was driven by Prof. Raffaele Mezzenga’s research group at ETH Zurich. Their fundamental breakthrough in amyloid fibril-carbon hybrid membranes has been successfully commercialized by the university spin-off company, BluAct Technologies, which has been scaled up for the highly efficient and sustainable removal of heavy metal ions, radioactive isotopes, organic pollutants, bacteria, and even viruses from contaminated water [98].

Meanwhile, PNFs serve as crucial components in hybrid composites for adsorption applications. While powdered MOFs show inherent limitations in contaminant adsorption due to their fragile crystalline structures and tendency to aggregate [99,100], incorporating β-lactoglobulin PNFs with UiO-66-NH_2_ into hybrid aerogels effectively addresses these challenges. In these composites, UiO-66-NH_2_ particles (80–100 nm) achieve uniform distribution throughout the matrix [59]. As safe and sustainable biomaterials, PNFs offer an innovative molecular engineering approach for enhancing traditional adsorbents. For example, exploiting the natural affinity between PNFs and carbon materials enables the creation of unique “rattan-like” three-dimensional macro-mesoporous membranes using carbon nanotubes. The BSA PNF surface modification creates highly porous structures with a multimodal pore-size distribution, thereby significantly improving mass-transfer characteristics and consequently enhancing adsorption rates and capacities (Fig. 4D) [90]. This PNF-based surface modification strategy can transform waste particles into valuable adsorbents. Through amyloid-mediated molecular engineering, PNF-modified particulate waste surfaces demonstrate 69–1980-fold higher adsorption capacities compared to established adsorbents, including ion exchange resins, activated carbon, covalent organic frameworks (COFs), and MOFs [101]. In conclusion, PNFs represent promising adsorbents owing to their inherent functional groups and residues (Table 2) [33,34,65,69,71,91,93,96,[102], [103], [104]]. Their safety profile, sustainability, and broad public acceptance have accelerated their development in environmental applications. PNFs can be engineered into adsorbents with diverse macrostructures, while their intrinsic properties enable the creation of hybrid composites with enhanced adsorption capacities and tailored functionalities. Furthermore, their exceptional adsorption capabilities and selective affinities make them valuable for surface modifications, broadening their potential applications.

**Table 2:** Overview of adsorption capacities of PNFs for various pollutants.

Types of protein	Composites	Macrostructure	Contaminants	Adsorption capacities (mg/g)	Ref.
β-lactoglobulin	-	-	Pb(Ⅱ)	212	[91]
Lysozyme	-	Membranes	Pb(Ⅱ)	270.3	[93]
Whey protein	Activated carbon	Membranes	Hg(Ⅱ)	25	[71]
BSA	-	Membranes	Au(Ⅲ)	1034.4	[103]
Lysozyme	-	Membranes	U(Ⅵ)	9.03	[96]
Lysozyme	g-C_3_N_4_	Nanosheets	Cr(Ⅵ)	23.5	[69]
β-lactoglobulin	-	Aerogel	Bisphenol A	50.6	[33]
			Bentazone	54.2	
			Ibuprofen	69.6	
Lysozyme	Magnetite	-	Reactive black 5	155	[104]
			Acid blue 29	101	
Whey protein	Carbon	Aerogels	Rhodamine B	125.2	[102]
β-lactoglobulin	ZIF-8	Aerogels	Hg(Ⅱ)	587	[34]
			Pb(Ⅱ)	161	
			Acid fuchsin	262.5	
			Crystal violet	202	
			Malachite green	220.7	
β-lactoglobulin	ZrO_2_	Membranes	F^−^	21.8	[65]

### 3.3 Synergistic advanced treatment for wastewater by protein nanofibrils

Adsorption processes alone cannot fully address water security concerns, as they merely concentrate contaminants into different phases rather than destroying them [13,105]. PNFs, however, extend beyond simple adsorption by serving as scaffolds or stabilizers for active nanoparticles and enzymatic catalysts. Their substrate characteristics, combined with exceptional adsorption properties, sustain catalyst activity. For instance, PNFs prepared from lysozyme, BSA, and ovalbumin deposited on carbon nitride surfaces expose hydrophobic alkyl and aryl groups, creating CO_2_ adsorption sites and enhancing mass transfer, which promotes reduction reactions while suppressing hydrogen production [106]. Additionally, PNF substrates regulate nanoparticle distribution, preventing aggregation and preserving catalytic activity. The bioorganic matrix functions as both “structure-directing” and “process-directing” agents, controlling the nucleation, growth, and morphology of inorganic materials. This control mechanism increases catalyst surface area, optimizes nanoparticle nucleation spacing, and enhances overall catalytic performance [107].

In the development of PNF-based hybrid composites, the ordered internal structure and robust trapping capacity for nanoparticles and enzymes enable the immobilization and stabilization of diverse catalysts, leading to enhanced and sustainable catalytic activities. For example, BSA PNF-based membranes demonstrate significant retention of horseradish peroxidase (HRP) and exhibit efficient phenol oxidation in the presence of 4-aminoantipyrine and H_2_O_2_. The catalytic activity increases proportionally with HRP loading, consistently outperforming free enzyme systems [55]. Similarly, lysozyme PNF biofilms enhance the stability of chloroperoxidase (CPO) composites, improving their reusability and maintaining high catalytic activity under harsh conditions [108]. The affinity of PNFs for metal ions creates additional opportunities for hybrid catalyst development. The integration of ZIF-8 and β-lactoglobulin PNFs into aerogels through freeze-drying yields materials with photocatalytic properties, enabling simultaneous adsorption and degradation of methylene blue. In these systems, zinc ions serve dual roles as ionic cross-linkers and catalytic centers, achieving up to 91.1% removal efficiency of hazardous dyes within 150 min of light exposure [34]. However, suboptimal catalytic performance may arise from preparation methods that compromise fibrillar structure, preventing effective catalyst distribution along the fibrils.

Maintaining the fibrous structure is crucial for achieving high catalytic activity in hybrid composite catalysts, where structural elements play multiple regulatory roles. In one study, gold nanoparticles synthesized in situ via NaBH_4_ reduction using β-lactoglobulin PNF templates showed remarkable efficiency in 4-nitrophenol (4-NP) reduction, exhibiting 1.5-fold and 3.0-fold higher activity compared to protein-templated and template-free Au nanoparticles, respectively. This enhanced performance stems from the assembled fibers directing Au nucleation, resulting in discrete Au nanofibers with an average particle size of 6.5 nm [109]. The in-situ deposition of metal nanoparticles demonstrates exceptional stability during catalysis. For instance, a catalytic film comprising alloy nanoparticle-loaded PNFs, developed for continuous-flow 4-NP reduction, maintained its integrity without detectable nanoparticle leaching through eight operational cycles [16]. Moreover, stable nanofibers form three-dimensional networks that enhance catalytic performance. β-lactoglobulin PNFs serving as templates for nano-Pd synthesis on carbon paper and titanium suboxide reactive electrochemical membranes achieved superior electroreduction efficiencies for Cr^6+^, 4-chlorophenol, and trichloroacetic acid compared to commercial cathodes. The three-dimensional PNF architecture enables higher Pd loading and increased electrochemically active surface area, with micro-/meso-scale pores providing active sites for electroreduction reactions, while macro-scale pores facilitate mass transport through enhanced convection [64].

**Fig. 5:**
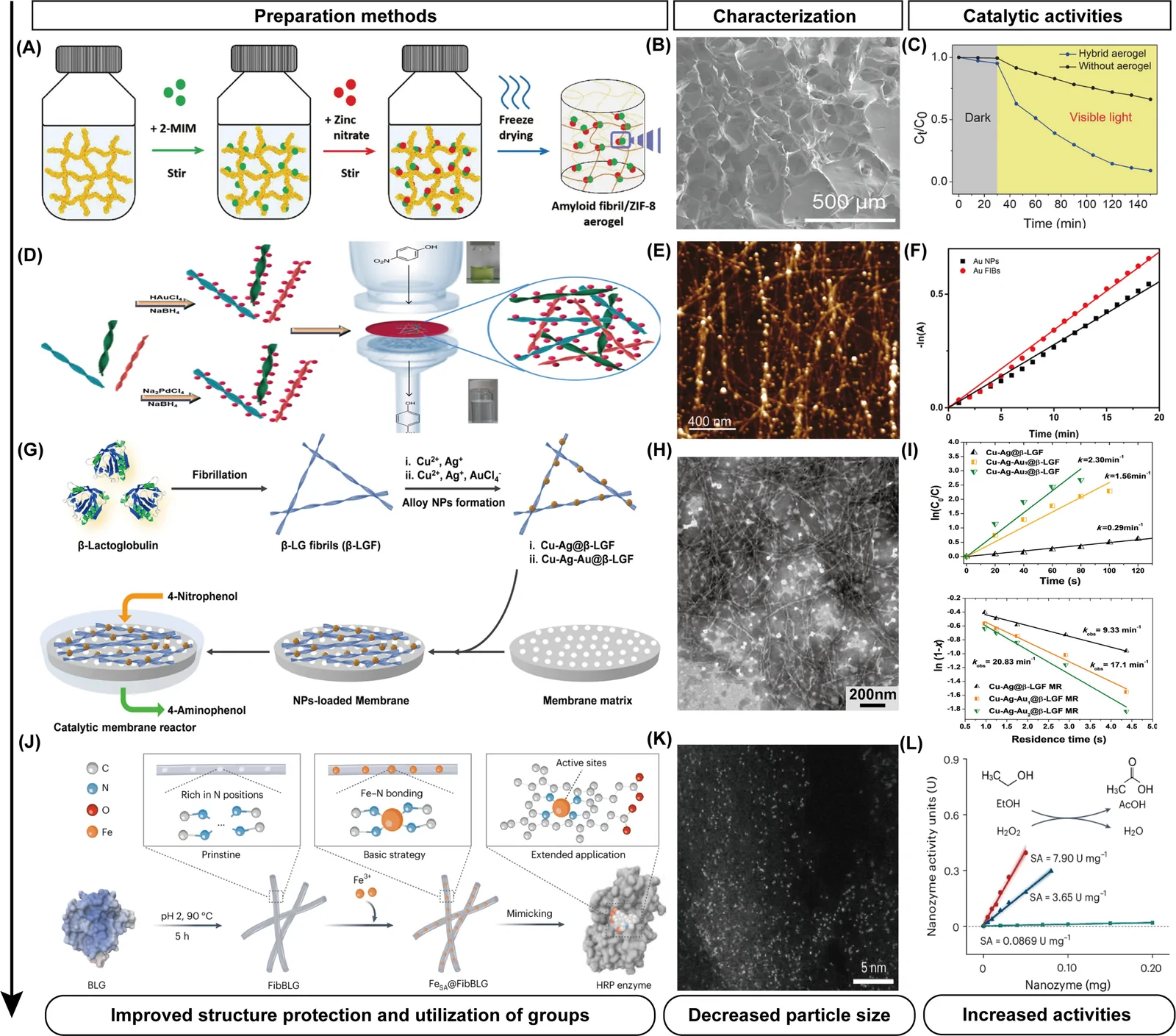
Overview of the preparation, characterization, and catalytic activities of diverse hybrid catalysts; (A−C) ZIF-8 crosslinked hybrid aerogels serving as photocatalysts, adapted from Ref. [34]. (D−F) Continuous catalytic membrane incorporating PNFs loaded with a single metal element, adapted from Ref. [36]. (G−I) Continuous catalytic membrane system utilizing PNFs loaded with multiple metal elements, adapted from Ref. [16]. (J−L) Single-atom catalyst construction via coordination between PNF nitrogen atoms and iron elements, adapted from Ref. [38]. Copyright American Chemical Society ([16,36]), Wiley-VCH ([34]) and Springer Nature ([38]).

The catalytic activity of PNF composites is primarily governed by two crucial parameters: active surface area and nanoparticle diameter. This relationship has been validated across various nanoparticles and preparation methods. Although both gold and palladium are commonly deposited onto PNF surfaces as catalytic nanoparticles, gold exhibits superior catalytic activity under comparable conditions. This enhanced performance likely stems from its larger surface area and reduced surface hindrance caused by amyloid fibril aggregation [36]. Preparation methods significantly influence nanoparticle size distribution on PNFs, thereby affecting catalytic performance. A comparative study of silver nanoparticles deposited on whey protein PNFs through chemical and photochemical reduction demonstrated distinct catalytic efficiencies in methylene blue reduction. Chemical reduction yielded complete conversion with lower activation energy, while photochemical reduction achieved only 60% conversion. This substantial difference was attributed to particle size distribution: chemical reduction produced nanoparticles predominantly below 10 nm, whereas photochemical reduction generated larger particles averaging 16 nm [110]. Metal reduction mediated by PNFs’ inherent functional groups can further minimize nanoparticle size and enhance catalytic activity. Research has shown that silver nanoparticles coordinated with carboxylic acid groups through electrostatic interactions form Ag cores along the nanofibers with remarkably small average particle sizes of 1.6 nm [111]. Similarly, coordinable nitrogen atoms can facilitate the formation of single-site iron nanozymes that selectively catalyze alcohol oxidation to acetic acid, demonstrating both efficacy and safety by reducing blood-alcohol levels by 55.8% [38]. In conclusion, PNFs serve as effective matrix materials for immobilizing enzymes and nanoparticles in catalytic reactions, significantly enhancing catalytic activities compared to systems lacking PNFs. These structures regulate catalytic performance through multiple mechanisms: mediating synthesis pathways, controlling particle size distribution, and providing expanded active reaction surfaces. As demonstrated in Fig. 5, catalytic activity is predominantly influenced by preparation methodology, which enables precise control over fibrous architecture and particle dimensions, ultimately optimizing active site availability and surface area for enhanced catalytic efficiency.

### 3.4 Surface modification for membrane separation through protein nanofibrils

Membrane separation technology has emerged as a promising approach for advanced water treatment, offering advantages of compact footprint, moderate energy consumption, and high contaminant removal efficiency [112]. This technology has demonstrated particular effectiveness in removing challenging pollutants, including extracellular antibiotic resistance genes and organic micropollutants through microfiltration systems [113,114]. Nevertheless, membrane fouling remains a significant challenge that limits broader application, although membrane modification presents an effective strategy for mitigating biofouling during preparation [115]. Recent advances in biomimetic approaches, drawing inspiration from natural materials, processes, and designs, have opened new possibilities for developing antifouling membranes [116]. PNFs represent ideal coating materials for membrane modification, offering unique advantages through their intrinsic surface adhesion properties and benign solution-based fabrication methods. These materials enable uniform coating across diverse surfaces, regardless of composition, size, shape, or structure, while maintaining high thermostability and mechanical robustness. Additionally, they exhibit substrate independence, resistance to organic solvents, and programmable functionalities [117,118]. Notably, β-lactoglobulin PNF-based multifunctional coating films demonstrate simultaneous control over surface nanostructure and chemical properties, achieving excellent performance in oil/water separation, antifouling, and antifogging applications [95]. This positions PNF coatings as a promising strategy for membrane modification to enhance water security.

**Fig. 6:**
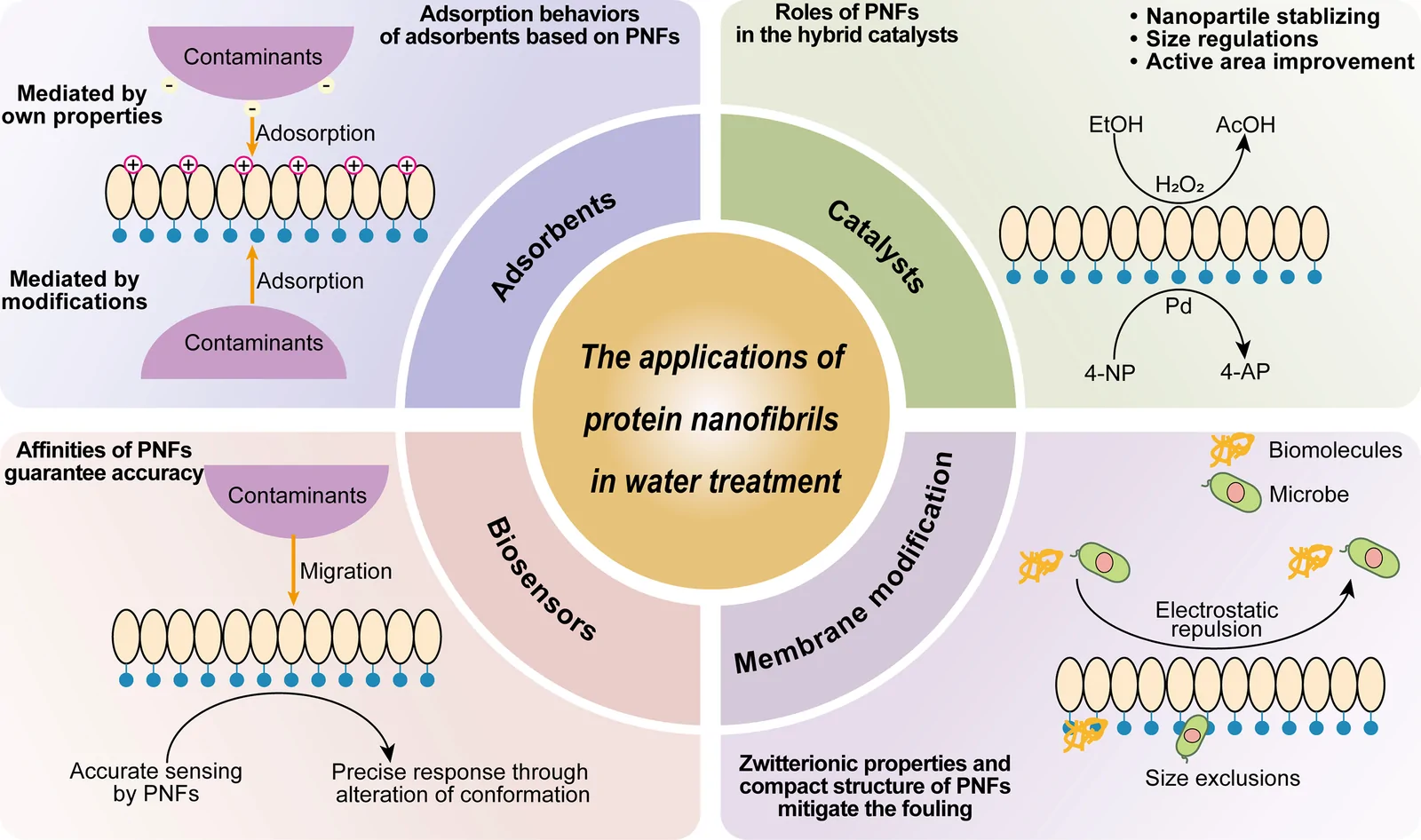
The summary of the PNF-based applications in chain-integrated water treatment, including interface detection, targeted enrichment, catalytic degradation, and contaminants separation.

Studies on PNF-based membrane modifications have primarily focused on utilizing their surface groups or combining them with other materials to resist biomolecular fouling. A notable example involves the development of phase-transited BSA PNF coating, achieved by immersing membranes in a protein-reductant mixture. This approach successfully preserved membrane porosity while maintaining microfiltration flux in dead-end filtration configurations. The resulting coatings demonstrated remarkable antifouling properties against both biomacromolecules (sodium alginate, humic acids, BSA) and microorganisms (*Sphingomonas wittichii* and *Aeromonas hydrophila*), primarily through electrostatic repulsion mechanisms [119]. This amyloid protein assembly-mediated surface modification has emerged as a versatile approach for functionalizing micro/nanoparticles across various compositions, dimensions, and morphologies [120]. For instance, in situ growth of ZIF-8 on phase-transited lysozyme PNF layer-modified polyethersulfone membranes yielded composites with exceptional antibacterial properties, achieving 99.9% efficiency against *E. coli* while maintaining resistance to organic fouling. These enhanced properties stem from the enrichment of hydrophobic amino acid residues and the net positive surface charge on the membrane. Additionally, ZIF-8 demonstrates photocatalytic activity, generating reactive oxygen species under solar irradiation [121]. While PNF-based membrane modifications and their composites offer property-dependent functionalities, with additional materials enabling features such as photocatalytic self-cleaning, some studies indicate potential impacts on membrane permeance [119,121]. However, comprehensive evaluation of PNF coating effects on baseline membrane performance remains limited. In summary, PNFs demonstrate exceptional potential in chain-integrated water treatment through their inherent multifunctional capabilities. Their intrinsic properties, including surface charge characteristics, polarity, and hydrophobic interactions, enable effective contaminant adsorption. Furthermore, their ability to integrate with functional materials expands their application scope, encompassing membrane modifications, catalytic degradation processes, and contaminant detection systems, as illustrated in Fig. 6.

## 4 Sustainable analysis

Given their high aspect ratios, a comparative analysis of PNFs, cellulose nanofibrils (CNFs), and carbon nanotubes (CNTs) was conducted to evaluate their practical potential [122]. Physical property analysis and life cycle assessments (Table S2) reveal that while both biological nanofibrils exhibit properties comparable to CNTs, they offer a substantially lower environmental impact. Although PNFs generally display lower mechanical strength than CNFs, their reported strength remains comparable to steel [123], underscoring their structural viability. However, Life Cycle Assessments (LCAs) highlight significant disparities in manufacturing energy and material costs. PNFs presented a decisive advantage over CNFs due to their energy-frugal fabrication, which relied on spontaneous self-assembly rather than energy-intensive mechanical disintegration. Furthermore, the economic viability of PNFs has been significantly enhanced by the recent transition from high-purity proteins to biomass waste precursors, effectively reducing costs [124,125]. Moreover, recent in-vitro and in-vivo evaluations have shown that digested fibrils cause no detectable cytotoxicity, physiological impairment, or organ-specific plaque accumulation, thereby establishing their safety equivalence to protein monomers [126].

Beyond sustainability, PNFs possess distinct biochemical advantages over traditional biomaterials like cellulose and chitosan. The diverse repertoire of 20 distinct amino acid side chains enables high-affinity molecular recognition through synergistic hydrophobic, electrostatic, and hydrogen-bonding interactions, which are largely absent in polysaccharide-based matrices [127]. Furthermore, PNFs offer superior architectural tunability; unlike the bulk chemical modification required for cellulose, the primary sequences of protein monomers can be genetically or enzymatically tailored. This precision allows for site-specific functionalization and the controlled assembly of hierarchical structures with predictable morphologies [128]. Collectively, these unique attributes, ranging from environmental compatibility and safety to versatile surface chemistry, position PNFs as superior candidates for advancing water quality management and sensing technologies.

## 5 Challenges and future perspective

### 5.1 Main challenges

Despite successful proofs-of-concept in pH and metal ion detection, PNF-based biosensors remain in a nascent stage of development. Their practical deployment is primarily hindered by complex environmental matrix effects. Specifically, the presence of co-existing ions induces charge screening, which diminishes the colorimetric response as ionic strength increases. Concurrently, fluctuations in water pH alter the electrostatic interactions between PNFs and contaminants, thereby compromising binding efficiency and sensor accuracy [83]. Beyond these matrix interferences, a fundamental challenge lies in the disparity between the trace concentrations of target contaminants and the high abundance of background species. Crucially, the limited selectivity of pristine PNFs, stemming from their reliance on non-specific interactions (e.g., electrostatic attraction and hydrogen bonding) rather than specific coordination geometries, means they lack the intrinsic capacity to discriminate between targets and ubiquitous background cations. These hurdles underscore an urgent need for robust strategies to mitigate interference and enhance detection reliability.

PNFs demonstrate effective contaminant adsorption capabilities through various macrostructures, notably as membranes and aerogels. However, fundamental challenges persist in the reproducibility of PNF performance and understanding their adsorption mechanisms. Firstly, unlike synthetic polymers, protein precursors often exhibit batch variations. Furthermore, the self-assembly of PNFs is highly sensitive to thermodynamic and kinetic parameters, leading to fibrils with distinct morphologies and surface properties [42]. The reported adsorption capacities of lysozyme-based PNFs for Pb^2+^ vary significantly across studies [92,93]. In addition, current research highlights the difficulty in determining specific amino acid-pollutant interactions and providing atomistic descriptions of adsorption processes, particularly in freeze-dried aerogel formations [33,89]. While freeze-drying techniques fail to maintain stable fibrous structures necessary for theoretical modeling, empirical evidence confirms the significant influence of fibrous architecture on adsorption behavior. Given that structural analysis of PNFs remains in its early stages, there is an urgent need to develop comprehensive analytical methods for characterizing PNFs derived from diverse raw materials.

In addition, the mechanical integrity of PNF-based macrostructures warrants careful consideration, as earlier research has highlighted the inherent fragility of pure PNF-derived amyloid films [37]. This structural instability often compromises functional performance; for instance, π−π interactions between PNFs and reduced graphene oxide can be disrupted during rigorous washing or electrochemical measurements in biosensing applications [85]. Furthermore, while PNFs serve as versatile matrices for enzymes and nanoparticles, their practical utility is frequently hampered by poor recyclability. Specifically, in peroxydisulfate activation systems, catalytic activity decreases markedly after five cycles, primarily due to the loss of active material in liquid-phase reactions [129]. Although incorporating catalytic centers as crosslinking agents has been explored to enhance stability, such systems often exhibit suboptimal efficiency [34], likely stemming from insufficient catalyst loading and compromised fibrous integrity. Collectively, these limitations underscore a pressing need to develop PNF-based catalytic systems that simultaneously achieve high efficiency and long-term structural stability.

### 5.2 Future perspective

Future research directions in PNF-based biosensors must address several key challenges to advance their practical implementation. While these biosensors have shown promise in contaminant detection, their universal applicability requires further validation across a broader range of target compounds. Although increasing PNF proportions can enhance biosensor accuracy [83], this approach does not fundamentally resolve interference issues. Functionalization of PNFs with nanoparticles can endow them with specific selectivity. In such hybrid systems, the nanoparticles serve as the active selective sites, while the PNF matrix effectively mediates their spatial distribution and particle size to ensure optimized performance [65]. Furthermore, the amino acid sequence and composition of PNFs can be rationally tailored via protein engineering to target specific contaminant properties. For instance, the intentional incorporation of additional positively charged residues can significantly enhance the adsorption of anionic pollutants through increased electrostatic affinity [130].

To address the challenges of performance reproducibility and structural integrity, two primary strategic directions have emerged. First, regarding fabrication, the observed performance discrepancies underscore an urgent need for standardized protocols and comprehensive reporting of synthesis conditions to ensure data reliability. Parallel to this, optimization of macrostructure preparation methods, such as vacuum-assisted membrane filtration and supercritical CO_2_ for aerogels, is essential to prevent structural collapse. Current structural analysis tools have provided valuable insights into PNF macrostructure, fundamental architecture, and intermolecular interactions [131]. However, these techniques show limitations in elucidating specific molecular forces and functional mechanisms critical for understanding binding behavior and functional group roles [132]. To bridge this gap, researchers are increasingly employing advanced structural and molecular biology tools to resolve outstanding questions about PNF structure-function relationships.

Addressing cyclic performance limitations requires constructing catalysts within stable macrostructures, such as membranes, hydrogels, and aerogels, which enhance nanoparticle stability. While PNF-based catalysts currently face challenges with limited nanoparticle binding sites, maintaining fibrous structure integrity can preserve protein residues and functional groups. Two promising strategies have emerged to protect fibrous properties: optimized crosslinker selection and macromolecular polymer incorporation. Recent work has demonstrated rapid synthesis of pure protein hydrogels through a protein unfolding-chemical coupling strategy, achieving formation within 10 min [133]. Furthermore, the introduction of macromolecular polymers enables the development of sophisticated double-network (DN) and triple-network (TN) gel systems. For instance, the successful integration of polyacrylamide with PNFs has yielded DN hydrogels that maintain fibrous characteristics [134].

To provide a holistic roadmap for the practical deployment of PNF-based technologies, we conducted a Strengths, Weaknesses, Opportunities and Threats (SWOT) analysis to evaluate their current standing and future trajectory in the water treatment sector. The primary strength of PNFs lies in their inherent biochemical diversity. Unlike conventional polysaccharides, the 20 different amino acids in PNFs provide a versatile platform for molecular recognition and specific binding of heavy metals and organic micropollutants [127]. However, certain weaknesses persist as critical bottlenecks, most notably structural instability. This is exemplified by the inherent fragility of pure PNF films and the limited recyclability of PNF-based composites [37,129]. As highlighted in recent literature [135], a major opportunity exists in their preparation cost, as PNFs could be produced from solid wastes, aligning with the circular economy goals [124,125]. However, the main threats involve the market dominance of established products, such as activated carbon and ion-exchange resins, coupled with the challenges of scaling up PNF production and preservation for industrial-scale adoption.

## CRediT authorship contribution statement

**Yiwei Liu:** Writing – original draft, Validation, Methodology, Investigation, Formal analysis, Conceptualization. **Zhengzheng Zhao:** Resources, Methodology, Investigation, Formal analysis, Data curation. **Yang Wu:** Writing – review & editing, Writing – original draft, Resources, Project administration, Methodology, Investigation, Funding acquisition, Formal analysis, Conceptualization. **Min Long:** Software, Resources, Methodology. **Xiong Zheng:** Writing – review & editing, Methodology, Investigation, Formal analysis, Data curation, Conceptualization. **Yinguang Chen:** Visualization, Supervision.

## Declaration of competing interests

The authors declare that they have no known competing financial interests or personal relationships that could have appeared to influence the work reported in this paper.
