# Supplementary material for: Engineered protein nanofibrils for chain-integrated water treatment: From source monitoring to end-point purification
Source: Eco Environ Health. 2026 Aug 24;5(3):100273. doi: 10.1016/j.eehl.2026.100273 (PMC13560018; doi:10.1016/j.eehl.2026.100273)
Supplement: Supplementary file 1 [file mmc1.docx]

**Supporting Information**

**Engineered protein nanofibrils for chain-integrated water treatment: From source monitoring to end-point purification**

Yiwei Liu ^a^, Zhengzheng Zhao ^a^, Yang Wu ^a,d,*^, Min Long ^a^, Xiong Zheng ^a,b,c,*^, Yinguang Chen ^a,c^

*^a^ State Key Laboratory of Water Pollution Control and Green Resource Recycling, College of Environmental Science and Engineering, Tongji University, Shanghai 200092, China*

*^b^ Key Laboratory of Yangtze River Water Environment, College of Environmental Science and Engineering, Tongji University, Shanghai 200092, China*

*^c^ Shanghai Institute of Pollution Control and Ecological Security, Shanghai 200092, China*

*^d^ Shanghai Key Laboratory of Urban Renewal and Spatial Optimization Technology, Shanghai 200092, China*

* Corresponding authors.

E-mail: [wuyang1026@tongji.edu.cn](mailto:wuyang1026@tongji.edu.cn) (Y. Wu); xiongzheng@tongji.edu.cn (X. Zheng)

**Figure S1** (A) Schematic diagrams of the detection mechanisms through PNF-based sensors adapted from [1]; (B) Key influencing factors for PNF-based detection platforms adapted from [2]. Copyright Wiley-VCH ([1]) and Elsevier ([2]).

**Table S1** Overview of PNF preparation methods

| **Materials** | **Methods** | **Conditions** | **References** |
| --- | --- | --- | --- |
| Lysozyme | Chaotropic agents | 50 ℃, 4 h | [3] |
| Lysozyme | Disulfide bond modification | TCEP addition | [4] |
| β-lactoglobulin | Acidic and high temperature | 90 ℃, 5 h | [5] |
| Lysozyme |  | 90 ℃, 24 h | [6] |

**Table S2** Comparative analysis of PNFs, CNFs, and CNTs: Physical properties, production, applications, and end-of-life management

| **Physical property** | **PNFs** | **CNFs** | **CNTs** | **References** |
| --- | --- | --- | --- | --- |
| Diameter | 5-10 nm | 5-100 nm | 0.4-100 nm | [7-10] |
| Length | Several micrometers | Several micrometers | Micros-centimeters |  |
| Size distribution | Polydisperse | Polydisperse | Depending on preparation |  |
| Tensile strength | 0.2-14 GPa | 2-4 GPa | 11-64 GPa | [11-15] |
| Young’s modulus | 3-20 GPa | 15-150 GPa | 0.27-1.47 TPa |  |
| Conductivity | None | None | 10^2^-10^8^ S/cm | [10, 13, 14, 16] |
| **Life cycle assessment** | | | | |
| Energy input | 7.5-36 kWht^-1^ | 500-2300 kWht^-1^ | 278000-250200000 kWht^-1^ | [7, 17] |
| Source | Natural sources | Natural sources | Fossil fuels | [7, 18] |
| Cost | $ 0-78.76/g | $ 2-3/g | $8-280/g | [9, 19] |
| Applications | Medicine, environment, energy and so on | Medicine, environment, energy and so on | Coating, films, biosensors, medicine and so on | [10, 20, 21] |
| Ecotoxicity | Low toxicity | Low toxicity | Potential challenges | [22-24] |
| Disposal | Biodegradable | Biodegradable | Persistent |  |

The energy input is according to energy consumption of heating during the preparation processes. The prices of protein raw materials are according to the price of Macklin Inc.

**References:**

[1] W.H. Leung, L. Zou, W.H. Lo, P.H. Chan, An amyloid-fibril-based colorimetric nanosensor for rapid and sensitive chromium(VI) detection, Chempluschem 78(12) (2013) 1440-1445. <https://doi.org/10.1002/cplu.201300267>.

[2] C. Kim, J. Park, W. Kim, W. Lee, S. Na, J. Park, Detection of Cd^2+^ and Pb^2+^ using amyloid oligomer–reduced graphene oxide composite, Bioelectrochemistry 147 (2022) 108214. <https://doi.org/https://doi.org/10.1016/j.bioelechem.2022.108214>.

[3] C. Liang, S.Z. Qin, H. Ai, S.S. Li, K.F. Du, Novel amyloid-like porous lysozyme skeletons as "green" superadsorbent presenting ultrahigh capacity and rapid sequestration towards hazardous Congo red, Chem. Eng. J. 441 (2022) 136005. <https://doi.org/10.1016/j.cej.2022.136005>.

[4] C. Li, L. Xu, Y.Y. Zuo, P. Yang, Tuning protein assembly pathways through superfast amyloid-like aggregation, Biomater. Sci. 6(4) (2018) 836-841. <https://doi.org/10.1039/c8bm00066b>.

[5] M. Peydayesh, M.K. Suter, S. Bolisetty, S. Boulos, S. Handschin, L. Nystrom, R. Mezzenga, Amyloid fibrils aerogel for sustainable removal of organic contaminants from water, Adv. Mater. 32(12) (2020) 1907932. <https://doi.org/10.1002/adma.201907932>.

[6] M. Peydayesh, T. Suta, M. Usuelli, S. Handschin, G. Canelli, M. Bagnani, R. Mezzenga, Sustainable removal of microplastics and natural organic matter from water by coagulation-flocculation with protein amyloid fibrils, Environ. Sci. Technol. 55(13) (2021) 8848-8858. <https://doi.org/10.1021/acs.est.1c01918>.

[7] D. Klemm, F. Kramer, S. Moritz, T. Lindström, M. Ankerfors, D. Gray, A. Dorris, Nanocelluloses: A new family of nature-based materials, Angew. Chem. Int. Ed. 50(24) (2011) 5438-5466. <https://doi.org/10.1002/anie.201001273>.

[8] R. Cabriolu, D. Kashchiev, S. Auer, Size distribution of amyloid nanofibrils, Biophys. J. 101(9) (2011) 2232-2241. <https://doi.org/10.1016/j.bpj.2011.09.053>.

[9] A.W. Carpenter, C.F. de Lannoy, M.R. Wiesner, Cellulose nanomaterials in water treatment technologies, Environ. Sci. Technol. 49(9) (2015) 5277-5287. <https://doi.org/10.1021/es506351r>.

[10] X. Ye, C. Lendel, M. Langton, R.T. Olsson, M.S. Hedenqvist, Protein nanofibrils: Preparation, properties, and possible applications in industrial nanomaterials, in: S. Thomas, Y. Grohens, Y.B. Pottathara (Eds.), Industrial Applications of Nanomaterials, Elsevier, Amsterdam, 2019, pp. 29-63.

[11] T. Saito, R. Kuramae, J. Wohlert, L.A. Berglund, A. Isogai, An ultrastrong nanofibrillar biomaterial: the strength of single cellulose nanofibrils revealed via sonication-induced fragmentation, Biomacromolecules 14(1) (2013) 248-253. <https://doi.org/10.1021/bm301674e>.

[12] I.A. Nyrkova, A.N. Semenov, A. Aggeli, M. Bell, N. Boden, T.C.B. McLeish, Self-assembly and structure transformations in living polymers forming fibrils, Eur. Phys. J. B 17(3) (2000) 499-513. <https://doi.org/10.1007/s100510070128>.

[13] T.W. Ebbesen, H.J. Lezec, H. Hiura, J.W. Bennett, H.F. Ghaemi, T. Thio, Electrical conductivity of individual carbon nanotubes, Nature 382(6586) (1996) 54-56. <https://doi.org/10.1038/382054a0>.

[14] M.S. Dresselhaus, G. Dresselhaus, J.C. Charlier, E. Hernández, Electronic, thermal and mechanical properties of carbon nanotubes, Philo. T. R. Soc. A. 362(1823) (2004) 2065-2098. <https://doi.org/10.1098/rsta.2004.1430>.

[15] T.P.J. Knowles, C.A. Waudby, G.L. Devlin, S.I.A. Cohen, A. Aguzzi, M. Vendruscolo, E.M. Terentjev, M.E. Welland, C.M. Dobson, An analytical solution to the kinetics of breakable filament assembly, Science 326(5959) (2009) 1533-1537. <https://doi.org/10.1126/science.1178250>.

[16] C.J. Chirayil, L. Mathew, S. Thomas, Review of recent research in nano cellulose preparation from different lignocellulosic fibers, Rev. Adv. Mater. Sci. 37(1-2) (2014) 20-28.

[17] M.F.L. De Volder, S.H. Tawfick, R.H. Baughman, A.J. Hart, Carbon nanotubes: present and future commercial applications, Science 339(6119) (2013) 535-539. <https://doi.org/10.1126/science.1222453>.

[18] L. Goldschmidt, P.K. Teng, R. Riek, D. Eisenberg, Identifying the amylome, proteins capable of forming amyloid-like fibrils, Proc. Natl. Acad. Sci. U. S. A. 107(8) (2010) 3487-3492. <https://doi.org/10.1073/pnas.0915166107>.

[19] Q.Q. Li, S. McGinnis, C. Sydnor, A. Wong, S. Renneckar, Nanocellulose life cycle assessment, Acs. Sustain. Chem. Eng. 1(8) (2013) 919-928. <https://doi.org/10.1021/sc4000225>.

[20] K. Aschberger, H.J. Johnston, V. Stone, R.J. Aitken, S.M. Hankin, S.A.K. Peters, C.L. Tran, F.M. Christensen, Review of carbon nanotubes toxicity and exposure-Appraisal of human health risk assessment based on open literature, Crit. Rev. Toxicol. 40(9) (2010) 759-790. <https://doi.org/10.3109/10408444.2010.506638>.

[21] J.A. Shatkin, T.H. Wegner, E.M. Bilek, J. Cowie, Market projections of cellulose nanomaterial-enabled products - Part 1: Applications, Tappi J. 13(5) (2014) 9-16. <https://doi.org/10.32964/tj13.5.9>.

[22] E.J. Petersen, L.W. Zhang, N.T. Mattison, D.M. O'Carroll, A.J. Whelton, N. Uddin, T. Nguyen, Q.G. Huang, T.B. Henry, R.D. Holbrook, K.L. Chen, Potential release pathways, environmental fate, and ecological risks of carbon nanotubes, Environ. Sci. Technol. 45(23) (2011) 9837-9856. <https://doi.org/10.1021/es201579y>.

[23] M. Vikman, J. Vartiainen, I. Tsitko, P. Korhonen, Biodegradability and compostability of nanofibrillar cellulose-based products, J. Polym. Environ. 23(2) (2015) 206-215. <https://doi.org/10.1007/s10924-014-0694-3>.

[24] M. Peydayesh, R. Mezzenga, Protein nanofibrils for next generation sustainable water purification, Nat. Commun. 12(1) (2021) 3248. <https://doi.org/10.1038/s41467-021-23388-2>.
